# Heavy Metal–Induced Hepatic Inflammation: Mechanistic Pathways, Epidemiological Evidence, and the Emerging but Unvalidated Role of Plant-Based Dietary Strategies

**DOI:** 10.3390/toxics14090793

**Published:** 2026-09-07

**Authors:** Jonah Bawa Adokwe, Phisit Pouyfung, Noriyoshi Ogino, Wantanasak Suksong, Supabhorn Yimthiang, Udomratana Vattanasit, Muttika Yongpraderm, Nuttapong Laemun, Yasuko Iwakiri, Tanaporn Khamphaya

**Affiliations:** 1 Health, Environment, and Safety Program, School of Public Health, Walailak University, Nakhon Si Thammarat 80160, Thailand; jonahbawa.ad@mail.wu.ac.th (J.B.A.); phisit.po@wu.ac.th (P.P.); ksupapor@mail.wu.ac.th (S.Y.); 2 Department of Occupational Health and Safety, School of Public Health, Walailak University, Nakhon Si Thammarat 80160, Thailand; muttika.yo@wu.ac.th; 3 Third Department of Internal Medicine, School of Medicine, University of Occupational and Environmental Health, Fukuoka 807-8555, Japan; n-ogino@med.uoeh-u.ac.jp; 4 Environmental Health and Technology Program, School of Public Health, Walailak University, Nakhon Si Thammarat 80160, Thailand; wantanasak.su@wu.ac.th (W.S.); udomratana.va@wu.ac.th (U.V.); 5 Department of Disease Control, Ministry of Public Health, Nonthaburi 11000, Thailand; laemunnn@gmail.com; 6 Section of Digestive Disease, Department of Internal Medicine, Yale School of Medicine, New Haven, CT 06510, USA; yasuko.iwakiri@yale.edu; 7 Excellence Center for Public Health Research, Walailak University, Nakhon Si Thammarat 80160, Thailand

**Keywords:** cadmium, lead, mercury, metabolic dysfunction-associated steatotic liver disease, hepatic inflammation, plant foods

## Abstract

Background: Chronic exposure to cadmium (Cd), lead (Pb), and mercury (Hg) has been associated with hepatic inflammation and metabolic liver dysfunction in experimental and epidemiological studies. Experimental evidence indicates that these metals can disrupt redox homeostasis, impair mitochondrial function, suppress nuclear factor erythroid 2-related factor 2 (Nrf2)-dependent antioxidant responses, and activate inflammatory signaling pathways, with potential relevance to metabolic dysfunction-associated steatotic liver disease (MASLD) and hepatocellular carcinoma (HCC). Methods: PubMed/MEDLINE, Scopus, Web of Science, and the Cochrane Library were searched for peer-reviewed English-language literature published from January 2000 to March 2026. Epidemiological, experimental, and interventional studies linking Cd, Pb, or Hg exposure to hepatic outcomes, as well as potentially relevant plant-derived bioactive compounds, were considered. Results: Human studies report associations between metal exposure and hepatic outcomes, although the evidence is largely observational and affected by important confounders. Polyphenols, flavonoids, alkaloids, and terpenoids show antioxidant, anti-inflammatory, and metal-chelating effects predominantly in vitro and in animal models, with limited clinical validation. Conclusions: Current evidence supports the liver as a potentially important target of chronic metal exposure; however, the available human evidence remains largely observational and does not establish causality. The hepatoprotective effects of plant-derived compounds remain predominantly preclinical and require validation in human populations. Future longitudinal and interventional studies should incorporate exposure assessment and appropriate control of confounding before clinical or dietary recommendations can be established.

## 1. Introduction

Heavy metals, namely cadmium (Cd), lead (Pb), and mercury (Hg), are pervasive contaminants that arise from geochemical processes and anthropogenic activities, including mining, industrial smelting, fossil fuel combustion, and the use of phosphate fertilizers [1,2,3,4]. They pose significant toxicological concerns because of their widespread distribution, high bioaccumulation potential, long biological half-lives, and established links to chronic organ dysfunction and cancer [5,6,7]. Unlike organic pollutants, these metals are non-biodegradable; as such, they persist indefinitely in the ecosystem and are biomagnified through food webs, resulting in human exposure in mining-affected and industrialized regions [8,9].

**Figure 1:**
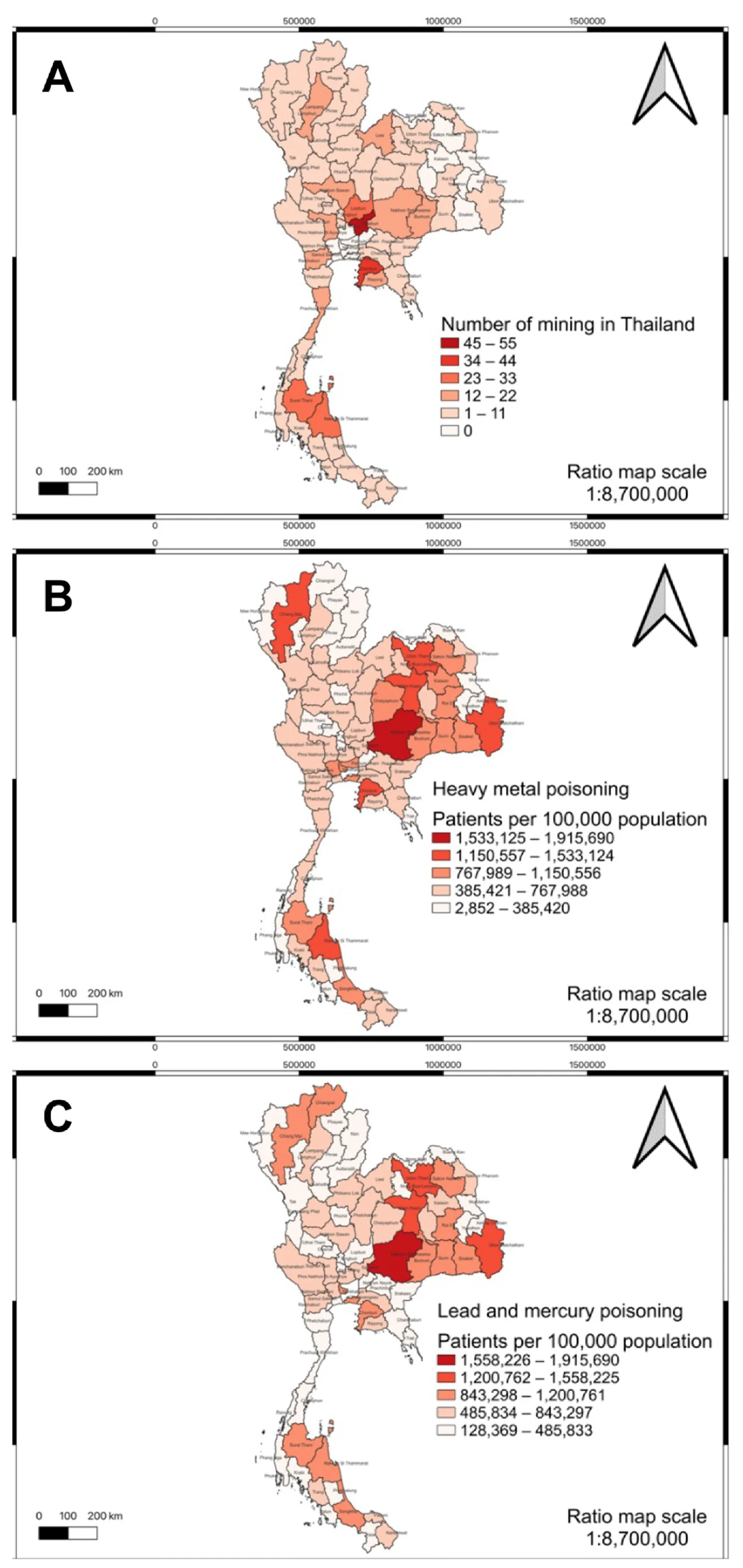
Geographic distribution of mining activities and heavy metal-related disease burden in Thailand. (**A**) Distribution of mining sites in Thailand. (**B**) Incidence of heavy metal poisoning (all types) by province (patients per 100,000 population). (**C**) Incidence of lead (Pb) and mercury (Hg)-specific poisoning by province (patients per 100,000 population). Darker shades of red indicate a higher disease burden. All maps include a standard scale bar at a ratio of 1:8,700,000. Data sources and processing: Mining site data were obtained from the Department of Primary Industries and Mines (DPIM), and disease incidence data were retrieved from the Health Data Center (HDC), Ministry of Public Health, Thailand [25]. Case definitions follow HDC coding for clinically reported heavy metal poisoning; provincial incidence was calculated as reported cases per 100,000 registered population, and no individual-level linkage between residence proximity and diagnosis was performed. Spatial visualization was performed using QGIS (version 3.40.5; QGIS Association, Switzerland). These maps are presented as descriptive context only. No formal spatial association analysis was performed; therefore, the geographic distributions shown should not be interpreted as evidence of a spatial or causal relationship between mining activities and disease burden.

This review focuses on Cd, Pb, and Hg because they are among the most prevalent environmental contaminants with established bioaccumulation potential and the largest body of hepatotoxicity literature. Other hepatotoxic metals (e.g., arsenic, chromium, nickel) are acknowledged but excluded to maintain focus, as their hepatic toxicology involves substantially different exposure contexts, speciation chemistry, and mechanisms that warrant separate treatment. Figure 1 depicts Thailand’s mining industry activities, which are concentrated in the central, western, and southern regions [10]. These maps are presented as descriptive context only; no formal spatial statistical analysis was performed, and spatial co-occurrence of mining activity and disease burden must not be interpreted as evidence of causation (ecological fallacy).

Populations near active or legacy mining operations, industrial facilities, and contaminated agricultural zones experience decades of disproportionate systemic metal accumulation at subacute doses, progressively impairing their organ function [2,11,12]. Traditionally, toxicological frameworks have prioritized organ-specific targets depending on the metal: nephrotoxicity and bone demineralization for Cd, and neurotoxicity for Pb and Hg [13,14]. Regulatory thresholds and occupational exposure limits rely heavily on these conventional endpoints. However, this “single chemical,” single-organ framing is increasingly recognized as inadequate for real-world mixture exposures, which can involve joint effects and convergence on shared biological pathways across levels of biological organization [15]. Emerging evidence suggests the liver may also be a sensitive, clinically relevant target, potentially affected at exposure levels below those associated with overt nephrotoxicity or neurotoxicity: heavy metal-induced oxidative stress, mitochondrial dysfunction, and inflammatory signaling promote chronic hepatic inflammation, disrupt lipid and metabolic homeostasis, and contribute to metabolic dysfunction-associated steatotic liver disease (MASLD) and hepatocellular carcinoma (HCC) development [16,17,18]. Collectively, these findings position heavy metals as plausible environmental contributors to liver disease, although this should not be interpreted as establishing a direct causal pathway in humans based on current evidence.

MASLD, formerly termed non-alcoholic fatty liver disease (NAFLD), is used throughout this review per current nomenclature [19]; metabolic dysfunction-associated steatohepatitis (MASH) replaces NASH where appropriate. The term NAFLD appears only when quoting or referencing older literature.

Given the widespread nature of chronic heavy metal exposure and limited global access to pharmaceutical chelation therapies, accessible and sustainable mitigation strategies are urgently needed. Plant-derived bioactive compounds, including polyphenols, flavonoids, alkaloids, terpenoids, and sulfur-containing compounds, exhibit multi-target hepatoprotective properties in experimental systems [20,21]. These phytochemicals are present in physiologically attainable concentrations in whole-plant foods, including cruciferous vegetables, berries, green tea, legumes, herbs, and grains. They exert coordinated antioxidant, anti-inflammatory, and metal-chelating actions that may directly counter heavy metal-induced hepatotoxicity [22,23]. Unlike isolated supplements, whole-plant food matrices provide complex mixtures of co-occurring bioactives, whose synergistic interactions may amplify their protective effects at dietary concentrations [17,24]. However, whether such effects occur at realistic dietary intakes in metal-exposed humans has not been demonstrated.

This review is guided by the hypothesis that the liver represents a clinically underappreciated target of chronic low-dose heavy metal toxicity, distinct from the classically emphasized organ-specific endpoints of heavy metal exposure. This review further evaluates the potential of plant-derived bioactive compounds in whole foods as accessible, multi-target strategies to mitigate metal-induced hepatic inflammation and associated metabolic dysfunctions. Integrating epidemiological, mechanistic, and translational evidence, this review examines the role of heavy metals in liver disease progression, including MASLD and HCC, while highlighting the possible dissociation between hepatic and conventional toxicity thresholds.

Accordingly, this review (i) characterizes the global epidemiological context of heavy metal exposure, with emphasis on mining-affected populations in Thailand and Asia; (ii) evaluates the evidence for molecular mechanisms by which Cd, Pb, and Hg induce hepatic inflammation, drive MASLD progression, and promote HCC development, with attention to the putative dissociation of hepatic and conventional toxicity thresholds; (iii) evaluates mechanistic and translational evidence for whole-plant food interventions as multi-target hepatoprotective strategies; and (iv) identifies research priorities and integrated clinical and public health considerations.

Most mechanistic insights derive from in vitro hepatocyte models and rodent studies employing acute or high-dose exposures that do not fully replicate chronic low-dose environmental conditions in humans; human data remain predominantly cross-sectional, limiting causal inference. Throughout this review, therefore, mechanistic descriptions are presented as experimentally established pathways, whereas human associations and mechanistic inferences without direct testing are interpreted as suggestive rather than definitive.

## 2. Materials and Methods

This narrative review used a structured literature search and evidence-selection approach to improve transparency and reproducibility. It was not designed as a systematic review or meta-analysis.

### 2.1. Literature Search Strategy

PubMed/MEDLINE, Scopus, Web of Science, and the Cochrane Library were searched for peer-reviewed English-language publications from January 2000 to March 2026. Search terms combined three concepts: exposure (“cadmium,” “lead,” “mercury,” or “heavy metal*”), hepatic outcomes (“liver,” “hepatotoxicity,” “hepatic inflammation,” “steatosis,” “MASLD,” “NAFLD,” “MASH,” “fibrosis,” or “hepatocellular carcinoma”), and dietary or biological modifiers (“plant*,” “phytochemical*,” “polyphenol*,” “flavonoid*,” “diet*,” or “food”). These three concept groups were combined in a single comprehensive search. Reference lists of relevant articles were also screened.

### 2.2. Eligibility Criteria and Study Selection

Epidemiological, experimental, clinical, and relevant review articles addressing cadmium, lead, or mercury exposure and hepatic outcomes were eligible. Studies unrelated to liver health, case reports, editorials, conference abstracts without full text, and non-peer-reviewed publications were excluded. Titles and abstracts were screened first, followed by full-text assessment by the author team. 

### 2.3. Data Extraction and Evidence Synthesis

Extracted information included study population, design, exposure metric and level, hepatic outcome, effect estimate and confidence interval, adjusted confounders, co-exposures, and major limitations. Evidence was synthesized narratively according to study type and strength. Quantitative estimates were reported descriptively and cross-checked against primary sources; meta-analysis was not performed because of substantial differences in populations, exposure measures, outcomes, and study designs.

## 3. Epidemiological Evidence and Hepatic Targeting of Heavy Metals

### 3.1. Environmental and Occupational Exposure Pathways

Heavy metals reach human populations through diverse routes beyond mining. Data on mining distribution (Figure 1A) indicate that mining activities are spatially clustered in specific provinces, with active lignite, metal ore, and metallurgical mining in the northern, central, and southern provinces exhibiting the highest contamination potentials [26,27,28]. However, the spatial patterns of the disease burden indicate a more complex exposure landscape. As illustrated in Figure 1B,C, heavy metal poisoning and Pb/Hg-related diseases are widely distributed across Thailand, extending beyond the major mining zones of the country. In the central, western, and southern regions, areas of higher mining density show spatial co-occurrence with areas of increased disease burden, consistent with global patterns of chronic low-dose exposure through environmental dispersion and bioaccumulation [10,26,27,28,29,30,31]. Importantly, this pattern does not fully apply to the northeast, where elevated disease rates were observed despite limited mining activity. This discrepancy suggests that mining is not the sole driver of exposure and that additional pathways contribute substantially to heavy metal exposure and the associated health risks [32,33]. Geographical co-occurrence of mining activity and disease does not establish causation.

Beyond mining, globally important exposure sources include electronic waste (e-waste) recycling, lead-acid battery recycling, informal smelting and manufacturing, industrial emissions, and contaminated food chains encompassing rice, leafy vegetables, fish, and shellfish [34]. In occupational settings, inhalation of metal-containing dust and fumes significantly affects workers in the mining, smelting, manufacturing, and informal industrial sectors [35,36]. Dominant pathways vary regionally: Cd exposure is linked to contaminated agricultural soils and phosphate fertilizers [37,38], and Pb and Hg exposure is linked to industrial emissions, informal recycling, and contaminated food chains [39,40]. Collectively, these pathways drive chronic low-dose exposure at the population level, facilitating systemic distribution and organ-specific accumulation, particularly in the liver.

### 3.2. Hepatic Accumulation and Low-Level Exposure

Following absorption, heavy metals are distributed systemically and accumulate in target organs. In Thailand, the metal body burden is reported to be two- to ten-fold higher in mining-prone areas, increasing with residence duration and reliance on locally produced food [41,42], with similar findings reported in mining-affected communities in China and other settings [43,44,45]. After gastrointestinal absorption, Cd, Pb, and Hg reach the liver via portal circulation, establishing it as an early accumulation site [18,46,47]; within hepatocytes, metals bind sulfhydryl-containing proteins such as metallothioneins and compartmentalize in mitochondria and lysosomes [48,49,50,51]. Hepatic retention differs substantially across metals: cadmium has a prolonged biological half-life (10–30 years); lead is predominantly stored in the bone, with only moderate and short-term hepatic retention [51]; and mercury accumulation depends strongly on chemical speciation, with organic methylmercury distributed more broadly than inorganic forms (Table 1) [52]. These differences underscore the need for caution when generalizing hepatic persistence across all three metals [53].

#### Hepatic Injury Relative to Conventional Thresholds

Traditional toxicological frameworks identify metal-specific conventional targets, and regulatory thresholds for Cd, Pb, and Hg are largely based on renal dysfunction, bone demineralization, and neurodevelopmental impairment [13,14,15,53,54,55,56]. Biomarker-based thresholds, such as urinary cadmium levels of ~5.0 μg/g creatinine, are widely used to define acceptable exposure limits [11,12,57]. However, accumulating evidence suggests that these thresholds may not adequately reflect hepatic risk. Experimental studies have demonstrated that Cd exposure below established renal toxicity thresholds can trigger early hepatic alterations, including nuclear factor kappa B (NF-κB) activation [58], elevated liver enzymes (ALT and AST), and lipid accumulation in cell-culture and rodent models [11,13]; the corresponding human evidence at comparable exposure levels remains observational (Table 2).

Current cross-sectional data raise the possibility that hepatic biomarker alterations may be detectable at exposure levels below established renal thresholds, though causality has not been demonstrated [18,59,60,61]. Evidence from Thailand and other exposed populations indicates higher metal burdens in individuals with liver dysfunction than in controls [17,62,63], consistent with, though not independently establishing, greater hepatic sensitivity to chronic low-dose exposure than previously recognized. Interpretation requires caution: common biomarkers (blood, urine, hair, toenail) reflect recent or cumulative exposure but do not directly represent hepatic metal burden [64]; most studies do not account for speciation or mixture co-exposure; and associations remain subject to confounding by alcohol use, smoking, obesity, metabolic disease, diet, and occupation. Well-designed longitudinal studies incorporating validated hepatic endpoints, metal speciation, and mixture-based analytical approaches are needed before any threshold difference can be considered established [65].

**Table 2:** Regulatory Reference Values and Toxicity Thresholds for Selected Heavy Metals: Renal Versus Hepatic Endpoints, with Co-Exposure and Speciation Considerations.

Heavy Metal	Regulatory Reference Value	Renal Threshold (Human)	Human Hepatic Effects (Low Exposure)	Experimental Hepatic Effect (Animal)	Metal Co-Exposure Effects	Chemical Speciation
Cadmium (Cd)	Urinary Cd: 5.0 μg/g creatinine (WHO, EU) [3,12]	Tubular proteinuria at ~2–5 μg/g creatinine [12,13]	Elevated ALT/AST and steatosis at <5 μg/g creatinine (cross-sectional) [12,18,60]	Liver injury at 0.5–2 mg/kg/day (rodent) [18]	Co-exposure with Pb shows supra-additive transaminase elevation in BKMR models; Cd is the dominant WQS-weighted contributor for MASLD risk [61,66]	The single ionic form, Cd^2+^, predominates.
Lead (Pb)	Blood Pb: 5 μg/dL (WHO clinical action level) [67]	Neurocognitive impairment > 5–10 μg/dL; CKD > 10–20 μg/dL	Elevated liver enzymes with blood Pb > 5–10 μg/dL [18,60]	Hepatotoxicity at 50–500 mg/kg (rodent) [18]	Supra-additive Cd × Pb interaction identified in BKMR models [41,60,68]	Predominantly inorganic Pb^2+^; organic forms now rare
Mercury (Hg)	Blood Hg: 5 μg/L (German HBM-I) [69]	Neurodevelopmental deficits at elevated Hg	Limited hepatic threshold data; insufficient for quantitative estimate [18]	Hepatotoxicity at 1–10 mg/kg (rodent) [18]	Three-way Cd × Pb × Hg interactions not yet adequately powered	Speciation-stratified threshold not yet established

Regulatory values are approximate and vary by agency. Human data are predominantly cross-sectional; findings represent associations rather than established causal thresholds. Confounding by alcohol use, obesity, and co-exposures cannot be excluded. Abbreviations: ALT, alanine aminotransferase; AST, aspartate aminotransferase; CKD, chronic kidney disease; WQS, weighted quantile sum regression; BKMR, Bayesian kernel machine regression.

### 3.3. Quantitative Exposure Response Evidence and Confounding

Clinical interpretation of biomarker elevations (Table 2). Where reported, the transaminase differences associated with these exposure levels are modest, typically on the order of a few units per liter ~3 to 4 U/L for ALT and ~2 U/L for AST and frequently remain within the conventional reference range [70]. The clinical significance of these modest transaminase elevations remains uncertain; they may represent subclinical injury or statistical noise, and their predictive value for progressive liver disease has not been established. Reporting relative risks without absolute effect magnitudes risks overstating clinical relevance. Findings from Thai mining-affected populations may not be directly generalizable to other global populations because exposure sources, exposure levels, nutritional status, and co-exposures may differ across settings. Occupational cohorts may also differ from environmentally exposed populations in exposure composition and intensity. Therefore, the quantitative findings summarized in Table 3 should be interpreted as population-specific observations rather than universally transferable thresholds.

### 3.4. Combined Metal Exposures, Speciation, and Nutritional Modifiers

Mercury-specific hepatic evidence is substantially more limited than for cadmium and lead; this evidence asymmetry should be considered when interpreting apparent differences among metals in the findings discussed below. Real-world Cd exposure rarely occurs in isolation. Co-exposure to Cd and Pb synergistically amplifies oxidative stress, disrupts nuclear factor erythroid 2-related factor 2 (Nrf2) signaling more potently than single-metal exposure, and accelerates transaminase elevation [36,38,59]. Weighted quantile sum regression (WQS) analyses consistently assign cadmium the largest weight within Cd/Pb/Hg mixtures for MASLD and liver enzyme outcomes, while BKMR models report a supra-additive Cd × Pb hepatic effect [60,68]. These findings reflect the mixture composition of the cohorts studied rather than a population-independent toxicity ranking, and available studies remain adequately powered only for two-way Cd × Pb terms; three-way Cd × Pb × Hg interactions have not yet been estimated.

#### 3.4.1. Metal-Specific Toxicokinetics and Differential Contribution to MASLD Stages

The three metals differ fundamentally in how they reach, persist within, and injure the liver, and these differences plausibly map onto distinct stages of MASLD progression [18,73,74]. Cadmium enters primarily through dietary ingestion, persists in hepatocytes for 10–30 years, and is the metal most consistently linked to chronic low-grade steatosis, fibrosis markers, and long-term hepatocellular damage, positioning Cd as the dominant candidate driver of slow steatosis-to-fibrosis progression. Lead disrupts Ca^2+^ signaling, is stored mainly in bone with only moderate, mobilizable hepatic retention, and produces episodic hepatic stress during redistribution; its hepatic contribution is therefore plausibly more acute-phase (transaminase fluctuation, inflammatory amplification) than fibrogenic. Methylmercury enters via L-type amino acid transporter 1 (LAT1) through amino-acid mimicry, is strongly neurotropic, and achieves hepatic concentrations far lower than in kidney and brain; its hepatic role, if any, is plausibly limited to oxidative amplification at high fish-based exposures. These remain working hypotheses, not demonstrated stage-specific effects.

#### 3.4.2. Metal–Essential Element Interactions

Nutritional status modifies mixture toxicity through direct transporter and binding competition. Iron deficiency upregulates divalent metal transporter 1 (DMT1) and Zrt- and Irt-like protein (ZIP) transporters, increasing Cd intestinal absorption and hepatic uptake [75,76]. Zinc sufficiency induces metallothionein synthesis, which competitively sequesters Cd and attenuates Cd-induced lipid peroxidation (Cd–Zn interaction) [49]. Calcium sufficiency reduces intestinal Pb absorption, and Pb mimics Ca^2+^ in intracellular signaling, so Pb–Ca competition modulates both uptake and downstream toxicity. Selenium binds Hg with very high affinity, forming biologically inert Hg–Se complexes and preserving selenoprotein antioxidant function (Hg–Se interaction); the molar Hg: Se ratio may be more informative than total Hg concentration [74,77]. Polyphenol-rich diets may further restore redox balance and enhance hepatic metal excretion [72,78]. Population-level hepatotoxicity cannot therefore be assessed without co-exposure profiles and baseline nutritional reserves.

#### 3.4.3. Mercury Speciation

Methylmercury biomagnifies through aquatic food webs, making fish and shellfish the dominant exposure route, and exploits amino acid transporters (LAT1, cysteine transporters) via methionine/cysteine mimicry [79]. Inorganic mercury, arising from artisanal gold mining, dental amalgam, and industrial processes, enters hepatocytes via DMT1 with intracellular handling through glutathione conjugation and ABC-transporter efflux. A single total-mercury threshold is therefore unlikely to be equally informative for hepatic risk across populations with differing speciation profiles; quantitative, speciation-stratified human hepatic-effect thresholds for mercury have not yet been established, an evidence gap reflected directly in the Table 2 mercury row.

### 3.5. Heavy Metals as Environmental Modifiers of MASLD and Prospective Evidence

MASLD is primarily driven by obesity, insulin resistance, and dyslipidemia. However, environmental exposure to heavy metals is increasingly recognized as a potential modifier of disease progression [61]. Epidemiological evidence from Thailand and other contaminated regions indicates that chronic exposure to multiple metals is associated with an increased risk of fatty liver disease and metabolic dysfunction, independent of traditional metabolic factors [66,68], and co-exposure to Pb and Cd has been linked to higher type 2 diabetes prevalence [41]. Experimental studies indicate that heavy metals interact with pathways underlying metabolic liver disease, including disruption of insulin signaling [75,80,81], promotion of *de novo* lipogenesis [82,83], impairment of mitochondrial β-oxidation [84], and activation of oxidative stress and inflammatory signaling [85].

Prospective evidence strengthens temporality but not causality. A 10-year Korean community cohort reported that baseline urinary cadmium (UCd) > 1.0 μg/g creatinine was associated with a 34% increased incidence of ultrasound-confirmed fatty liver disease, independent of metabolic syndrome components [71]; a prospective US analysis associated higher baseline blood cadmium (BCd) with accelerated progression from simple steatosis to fibrotic MASLD over 5 years [17,60]; and cohort studies adjusting for alcohol, viral hepatitis, and BMI reported 2.1–3.4-fold hazard ratios for cirrhosis-related mortality in the highest UCd quartile [72,86]. Even adjusted prospective estimates cannot fully exclude residual confounding by unmeasured dietary, occupational, or genetic factors. Reverse causality is unlikely for metal-to-liver directions given metals’ temporal precedence, but metabolic disease can alter metal handling (e.g., altered renal Cd clearance), complicating interpretation. Exposure mixtures, selection bias in cohort enrollment, and biomarker misclassification further limit inference. Hepatic toxicity thresholds have not been firmly established in humans.

## 4. Molecular Mechanisms of Heavy Metal-Induced Hepatic Injury

### 4.1. Hepatocellular Uptake: Transport Proteins and Speciation-Dependent Pathways

The initial cellular entry of toxic metals into hepatocytes is a critical rate-limiting step in hepatic accumulation and subsequent toxicity. Cd, Pb, and Hg exploit specific membrane transporters and endocytic pathways, often mimicking essential physiological substrates [87,88].

#### 4.1.1. Divalent Metal Transporters

Free ionic Cd^2+^ and Pb^2+^ exploit the physiological machinery of essential divalent cations, particularly Fe^2+^ and Ca^2+^ [89]. The primary sinusoidal conduit is DMT1 (SLC11A2), a proton-coupled symporter experimentally validated to transport Cd^2+^ and Pb^2+^ with high affinity [88,90]; DMT1 expression increases under dietary iron deficiency, accelerating toxic metal uptake. ZIP8 (SLC39A8) and ZIP14 (SLC39A14) serve as major Cd^2+^ entry pathways [76] and are inducible by inflammatory cytokines (Interleukin 6, IL-6; tumor necrosis factor alpha, TNF-α), so that metal-induced hepatic inflammation dynamically upregulates its own uptake machinery, a feed-forward loop with implications for chronic low-dose exposure [91]. Transient receptor potential cation channel subfamily V member 6 (TRPV6) provides a calcium-mimetic Cd^2+^ route, and Pb^2+^ shows affinity for voltage-gated calcium channels, though their hepatocellular contribution remains secondary to DMT1 [92].

#### 4.1.2. Speciation-Dependent Uptake and the Revised Protein-Binding Model

**Figure 2:**
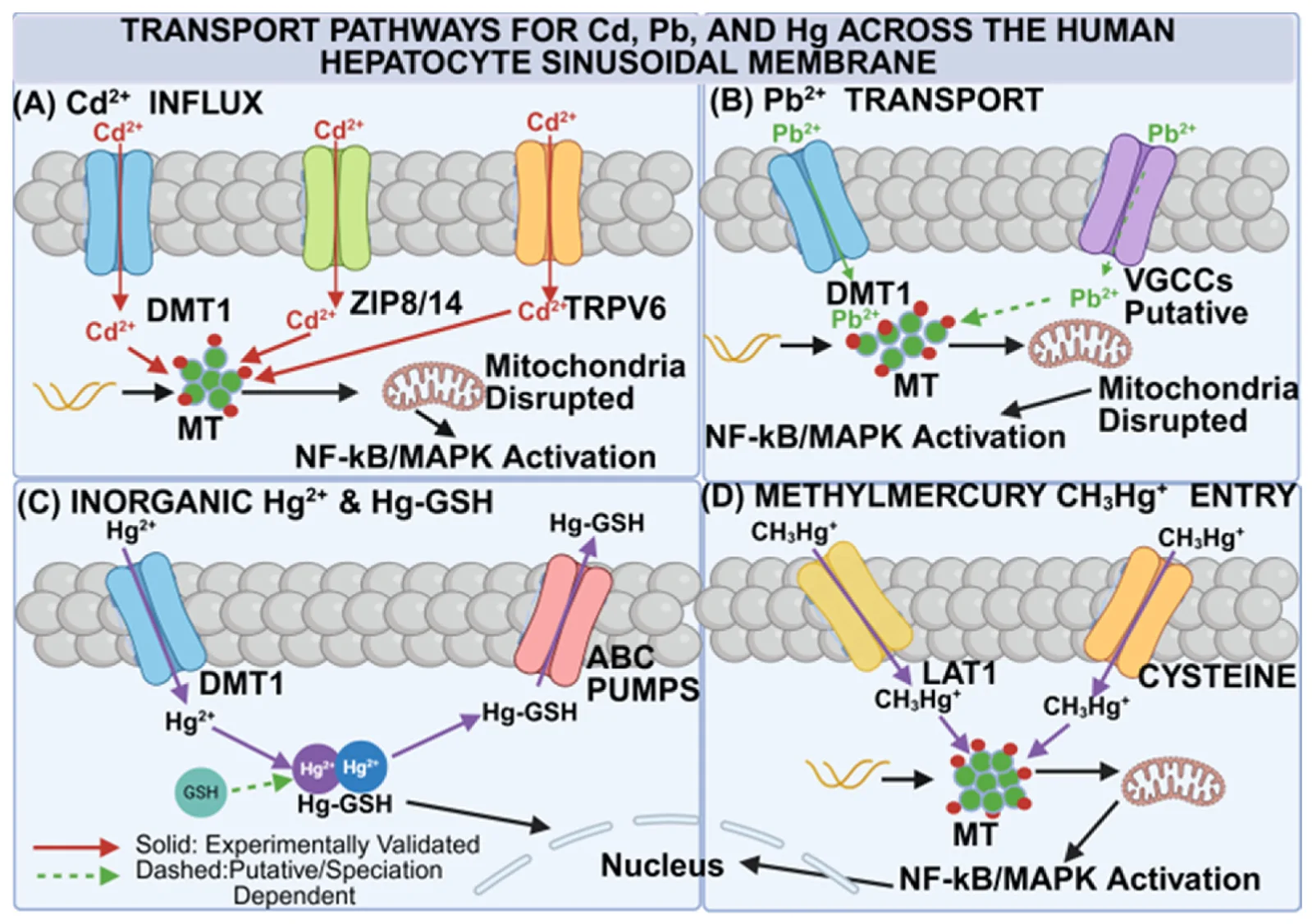
Transport pathways for Cd, Pb, and Hg across the sinusoidal membrane of the human hepatocyte. (**A**) Cd^2+^ influx via DMT1, ZIP8/14, and TRPV6. (**B**) Pb^2+^ transport via DMT1 and potential VGCCs. (**C**) Inorganic Hg^2+^ via DMT1 and Hg-GSH complex handling via ABC efflux pumps. (**D**) Methylmercury (CH3Hg+) entry via LAT1 and cysteine transporters through amino acid mimicry. Intracellularly, metals are sequestered by MT, disrupt mitochondria, and activate NF-κB/MAPK pathways. Hepatocytes primarily accumulate free ionic Cd^2+^ via transporter-mediated uptake; unlike renal proximal tubule cells, hepatocytes do not internalize Cd bound to albumin or β2-microglobulin via endocytosis [88,95]. Solid arrows = experimentally validated transport routes demonstrated in peer-reviewed studies; dashed arrows = putative or speciation-dependent pathways inferred from indirect evidence or analogy. Arrow colors indicate metal-specific pathways: red, Cd; green, Pb; and purple, Hg; black arrows indicate downstream intracellular processes.

In systemic circulation, metals exist predominantly as protein-bound complexes. Recent critiques of the classical Nordberg model [88,93] demonstrate that plasma metallothionein concentrations (0.7 mM) and other carriers [94]; that albumin binding reduces rather than enhances free Cd^2+^ bioavailability, with hepatocyte uptake correlating strictly with the unbound fraction [95]; that hepatocytes, unlike renal proximal tubule cells, lack megalin/cubilin-mediated endocytosis of Cd–protein complexes [88,95]; and that metallothionein (MT)-null mice exhibit severe Cd toxicity, proving intracellular MT is protective storage rather than a toxic transport vehicle [94]. We therefore retain the revised model: (1) free Cd^2+^ influx via DMT1, ZIP8/14, and TRPV6; (2) albumin as a plasma buffer limiting free Cd^2+^ availability; (3) chronic overload and inflammatory signaling upregulating ZIP8/14 and DMT1; (4) speciation-dictated Hg handling inorganic Hg^2+^ via DMT1 with Hg–glutathione export through ABC transporters, methylmercury via LAT1 and cysteine transporters (Figure 2) [96].

### 4.2. Mitochondrial Dysfunction, Oxidative Stress, and Nrf2 Suppression

**Figure 3:**
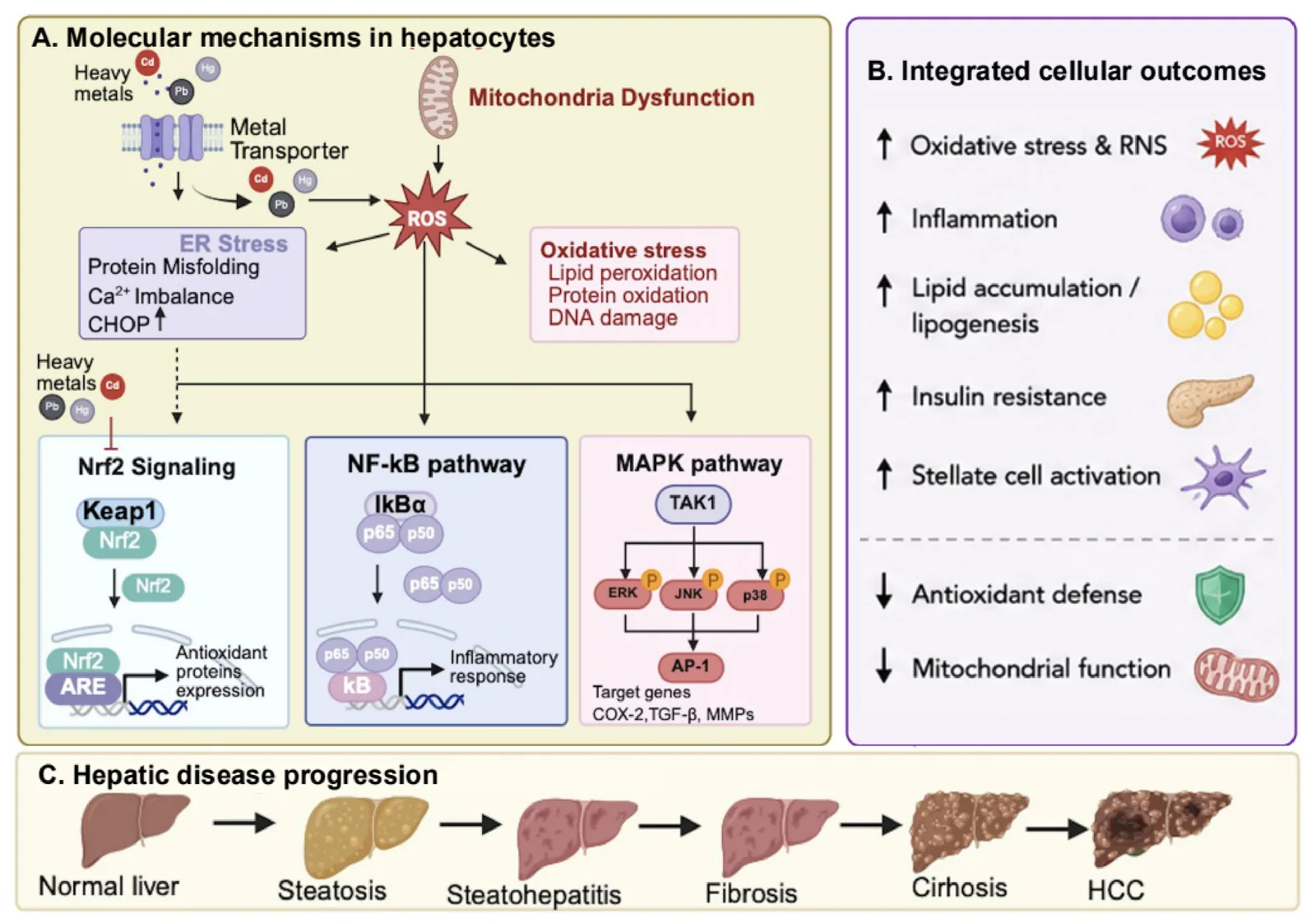
Molecular mechanisms and pathological progression of heavy metal-induced hepatotoxicity. (**A**) Heavy metals disrupt hepatocyte homeostasis by inducing mitochondrial dysfunction, endoplasmic reticulum stress, and oxidative stress, while dysregulating Nrf2, NF-κB, and MAPK pathways. (**B**,**C**) These molecular insults drive adverse cellular outcomes including inflammation, lipid accumulation, and stellate cell activation that propel the progressive continuum of liver disease from normal tissue through steatosis, steatohepatitis, and fibrosis, ultimately culminating in cirrhosis and hepatocellular carcinoma (HCC).

Mitochondrial dysfunction initiates heavy metal-induced hepatotoxicity in experimental models, triggering downstream oxidative stress and inflammatory signaling [97]. Cd and Hg directly interfere with electron transport chain complexes I and III, causing electron leakage and excessive reactive oxygen species (ROS) production [84,98]. Pb disrupts mitochondrial calcium homeostasis and membrane potential, impairing the electrochemical gradient required for ATP synthesis [99]. Hg, with high thiol affinity, additionally inhibits cytochrome oxidase and succinate dehydrogenase [73,97]. These metal-specific effects converge on impaired bioenergetics, reduced ATP availability, and ROS overproduction that damages lipids, proteins, and nucleic acids [97,100]. Direct translation to chronic low-dose human exposure remains uncertain. Sustained ROS overproduction disrupts the Nrf2 antioxidant defense pathway [101,102]: cadmium promotes Keap1-dependent Nrf2 degradation, while mercury directly modifies the cysteine residues required for Nrf2 activation [79,84,98,101], decreasing antioxidant enzyme expression and closing a self-amplifying loop that sensitizes hepatocytes to inflammatory activation (Figure 3).

### 4.3. Inflammatory and Immune Signaling: NF-κB, MAPK, and Beyond Hepatocytes

The combined mitochondrial and oxidative stress signal activates nuclear factor kappa B (NF-κB), which is highly redox-sensitive [50,80]. Heavy metals promote inhibitor of nuclear factor kappa B alpha (IκBα) phosphorylation and degradation, enabling NF-κB nuclear translocation and transcriptional activation of TNF-α, IL-6, and interleukin 1 beta (IL-1β) [23,61]. Cadmium potently activates NF-κB directly in hepatocytes [103], whereas lead and mercury preferentially enhance NF-κB activation in non-parenchymal cells [58,104]. Parallel mitogen-activated protein kinase (MAPK) pathways (JNK, p38, ERK) amplify this signal, with metal-specific preferences: cadmium strongly activates JNK and p38; lead preferentially modulates ERK/p38; mercury activates all three dose-dependently [98,105,106]. NF-κB and MAPK cross-talk sustains a positive feedback loop under chronic low-dose exposure [58], connecting inflammation to MASLD progression via TNF-α-mediated insulin resistance and lipogenesis [75,80,107] with innate immune pathways, including cGAS/STING/NF-κB increasingly implicated in MAFLD pathogenesis [108] and to HCC via IL-6/TNF-α-driven proliferation and immune escape [38,109], consistent with emerging evidence on NASH-to-hepatocellular carcinoma progression (Figure 3) [110].

#### Non-Parenchymal Cells and Intercellular Crosstalk

Hepatic inflammation and fibrosis are multicellular processes; a hepatocyte-only view is incomplete. Kupffer cells. Experimental studies indicate that Cd exposure promotes ROS-dependent NF-κB activation in Kupffer cells, driving secretion of TNF-α, IL-6, and monocyte chemoattractant protein-1 (MCP-1) [50,60,103], which recruit circulating immune cells and sustain chronic inflammation. Metal-induced oxidative stress and Kupffer-cell cytokines activate Hepatic stellate cells (HSCs); transforming growth factor beta (TGF-β)-driven trans differentiation into myofibroblasts initiates collagen deposition and fibrogenesis, establishing a pro-tumorigenic microenvironment [84,104,111,112]. Liver sinusoidal endothelial cells (LSECs). LSECs regulate sinusoidal perfusion and immune tolerance; their capillarization and barrier dysfunction accompany chronic injury and may be perturbed by metal-induced oxidative stress [104,113].

Cholangiocytes. Biliary epithelium is exposed to metals excreted in bile; cholestatic and biliary injury patterns have been described in metal-exposed experimental systems [114]. Infiltrating immune cells. Monocyte-derived macrophages and neutrophils recruited via MCP-1 and other chemokines amplify injury and shape fibrosis resolution or progression [115]. Paracrine crosstalk among these populations- hepatocyte-derived damage signals activating Kupffer cells, Kupffer-derived cytokines activating HSCs, and LSEC dysfunction altering immune recruitment converts acute injury into self-sustaining chronic inflammation and is the cellular substrate on which the emerging regulated cell-death pathways described next act [115].

### 4.4. Emerging Molecular Pathways in Metal-Induced Hepatic Injury

Beyond oxidative stress and canonical inflammation, experimental studies have implicated several emerging pathways in metal-induced hepatic injury [18]. Ferroptosis. Heavy metals promote iron-dependent lipid peroxidation and can inhibit glutathione peroxidase 4 (GPX4), the central ferroptosis defense enzyme, by depleting glutathione or disrupting iron homeostasis; Cd-induced hepatotoxicity in rodent models shows hallmarks of ferroptosis (lipid ROS accumulation, labile iron elevation) that are rescuable by ferrostatin-1 or iron chelation [18]. Pyroptosis and the NLR family pyrin domain containing 3 (NLRP3) inflammasome. Metal-induced ROS and mitochondrial damage activate the NLRP3 inflammasome, driving caspase-1-mediated cleavage of gasdermin D and release of IL-1β and IL-18, propagating sterile inflammation in Kupffer cells and hepatocytes [83]. 

Necroptosis. Receptor-interacting protein kinase 1 (RIPK1)/receptor-interacting protein kinase 3 (RIPK3)/mixed lineage kinase domain-like protein (MLKL)-mediated necroptosis has been implicated in metal-induced hepatocyte death under conditions where apoptosis is blocked, contributing to inflammatory amplification through release of damage-associated molecular patterns [116,117]. Autophagy and mitophagy. Cd and Hg impair autophagic flux and selective clearance of damaged mitochondria (mitophagy), so that dysfunctional, ROS-generating mitochondria accumulate, compounding oxidative injury; whether this reflects blockade of lysosomal degradation by metal-accumulated lysosomes is an active area of investigation. Endoplasmic reticulum stress. Cd and Pb activate the unfolded protein response (protein kinase R-like endoplasmic reticulum kinase, PERK; inositol-requiring enzyme 1 alpha, IRE1α; activating transcription factor 6, ATF6); prolonged ER stress intersects with NF-κB signaling and promotes lipogenesis and apoptosis, linking metals to metabolic liver injury [80]. Gut–liver axis. Oral metals alter gut microbiome composition and barrier integrity in animal models, modifying bile acid metabolism and portal endotoxin delivery; dysbiosis-driven hepatic inflammation represents a plausible amplification route for dietary Cd and a mechanistic rationale for fiber- and phytochemical-rich diets [116].

### 4.5. Epigenetic Regulation

Heavy metals alter the epigenetic landscape of hepatic cells. Cadmium induces CpG island hypermethylation and silencing of tumor suppressor genes, alters histone acetylation and methylation patterns, and dysregulates microRNAs, including upregulation of pro-inflammatory/pro-fibrotic miR-21 and miR-155 and perturbation of the hepatocyte-enriched miR-122, thereby modulating inflammatory signaling, apoptosis, and carcinogenic susceptibility [118]. These modifications are heritable across cell divisions and may partially explain persistent injury after exposure reduction, though human validation is lacking.

### 4.6. Progression to Hepatocellular Carcinoma

Chronic exposure to heavy metals contributes to hepatocarcinogenesis through sustained inflammatory signaling, oxidative stress, genomic instability, and immune dysregulation, as demonstrated in experimental models and supported by emerging epidemiological evidence. Cadmium (International Agency for Research on Cancer (IARC) Group 1) promotes hepatocarcinogenesis through ROS-driven genotoxicity, epigenetic silencing, and chronic NF-κB/IL-6 inflammation with PI3K/AKT-mediated survival signaling [91,99,100,101,102]. Lead (Group 2A) generates oxidative DNA strand breaks, inhibits repair, and drives TGF-β-mediated stellate cell activation and fibrogenesis [99,105,119,120]. Mercury (Group 2B) inhibits thiol-binding enzymes and generates oxidative DNA damage, but epidemiological data for HCC risk remain limited and inconclusive (Table 4) [98,121,122].

### 4.7. Novel Biomarkers for Subclinical Metal-Induced Liver Injury

Conventional ALT/AST lack sensitivity for subclinical injury and fluctuate within the normal range. Several newer markers may improve detection of metal-associated hepatic injury below current thresholds [57,125], miR-122, a hepatocyte-enriched microRNA released early in hepatocyte injury and already noted as epigenetically perturbed by metals [126]; glutamate dehydrogenase (GLDH), a55 mitochondrial matrix enzyme indicating mitochondrial-specific damage; cytokeratin-18 fragments (CK-18 M30, apoptosis-specific, and M65, total cell death), validated in MASLD trials; transient elastography (FibroScan; Echosens, Paris, France), providing non-invasive liver stiffness for fibrosis staging; and MRI-proton density fat fraction (MRI-PDFF), quantifying hepatic steatosis with high reproducibility. Ongoing Southeast Asian and European biomonitoring cohorts are incorporating serial elastography and metabolomics. None of these markers has yet been validated specifically for metal-exposed populations, and their adoption for surveillance in such populations remains a research priority rather than clinical practice.

## 5. Plant-Based Dietary Interventions: Preclinical Evidence and Translational Limitations

### 5.1. Preclinical Evidence for Phytochemicals

All evidence presented in this section derives from preclinical models (in vitro hepatocyte assays and metal-exposed rodent studies) and does not constitute proof of efficacy in humans. No randomized controlled trial of a plant-based dietary intervention in a metal-exposed population has been identified in the literature reviewed here. Heavy metal-induced hepatic injury involves interconnected disturbances in mitochondrial bioenergetics, oxidative balance, inflammatory signaling, metabolic regulation, and immune homeostasis, necessitating multi-target rather than single-pathway interventions [59,109]. In experimental systems, plant-derived compounds modulate the integrated oxidative-inflammatory cascade established in Section 4.2, Section 4.3 and Section 4.4 rather than acting through entirely distinct mechanisms. Major bioactives include polyphenols, flavonoids, alkaloids, terpenoids, and sulfur-containing compounds [22], present at physiologically attainable levels in whole foods, a claim that Section 5.2 substantially qualifies. Mechanistic and food-source evidence are combined in Table 5. In long-term metal exposure models, flavonoid supplementation prevents hepatic lipid peroxidation, inflammatory infiltration, and fibrosis markers; berry anthocyanins attenuate oxidative injury and lipid accumulation in HepG2 cells [71,127,128]; cruciferous vegetables exert hepatoprotection via glucosinolates and hydrolysis products such as sulforaphane and indole-3-carbinol, which enhance phase II detoxification (glutathione S-transferase, GST; NAD(P)H quinone dehydrogenase 1, NQO1) [129], green tea catechins (epigallocatechin gallate, EGCG; epicatechin gallate, ECG) and herbs (curcumin, allicin, gingerol) modulate the shared oxidative-inflammatory network alongside AMP-activated protein kinase (AMPK)-mediated metabolic regulation [23,128,130]. Observational data from metal-exposed populations associate higher cruciferous and polyphenol-rich dietary intake with improved detoxification capacity, lower circulating metal concentrations, and improved antioxidant status [72,78,131], but these human data are cross-sectional and do not validate the preclinical mechanisms.

Three distinctions govern interpretation of this evidence. First, isolated compounds at experimental doses (typically tens to hundreds of mg/kg in rodents, or micromolar concentrations in cell culture) must be distinguished from realistic dietary intake; human-equivalent conversions of effective preclinical doses frequently exceed what whole-food diets can supply. Second, bioavailability differs fundamentally: purified compounds applied to cell culture or administered as extracts bypass the first-pass intestinal and hepatic metabolism that whole-food consumption entails, so effective concentrations in vitro may never be reached systemically from food. Third, the whole-food synergy hypothesis that co-occurring phytochemicals act synergistically remains plausible but untested against an isolated-compound comparator under matched dosing in any metal-exposed model. Translational challenges additional to dose and bioavailability include long-term safety of high phytochemical intakes, inter-individual variation in microbiome-mediated metabolism of polyphenols, and the near-total absence of clinical intervention studies in metal-exposed populations.

### 5.2. Translational Limitations

The mechanistic evidence summarized in Section 5.1 derives almost entirely from in vitro hepatocyte assays and rodent models employing isolated phytochemicals at doses far exceeding chronic environmental human exposure. Rodent hepatoprotection studies typically administer purified compounds at tens to hundreds of milligrams per kilogram, alongside metal challenge doses that are themselves orders of magnitude above real-world exposure, since both the toxic insult and the protective compound are tested under supraphysiological conditions simultaneously. Human-equivalent dose conversions are rarely reported, and where available for compounds such as curcumin or EGCG, they frequently correspond to intakes exceeding what whole-food diets can realistically supply. The proposed synergistic effects of whole-food phytochemicals remain hypothetical and have not been directly validated in metal-exposed human populations. Most cited studies test isolated, purified phytochemicals applied directly to cell culture or as concentrated extracts, bypassing extensive first-pass metabolism. Moreover, the long-term safety of phytochemical supplementation in metal-exposed populations remains insufficiently established. Finally, no human randomized controlled trial evaluating a plant-based dietary intervention specifically in a metal-exposed population has been identified; available clinical evidence derives from MASLD populations selected for metabolic disease status, not documented heavy metal exposure. Several studies failed to demonstrate significant hepatoprotection following polyphenol supplementation under chronic low-dose exposure conditions. Taken together, the hepatoprotective claims in Section 5.1 should be regarded as mechanistically plausible hypotheses generated from preclinical systems, not as findings with demonstrated relevance to chronic, real-world human exposure.

## 6. Discussion

### 6.1. Integrated Discussion

The three metals differ meaningfully in hepatotoxic profile. Cadmium, ingested primarily through diet and retained hepatically for 10–30 years, shows the most consistent human associations with hepatic endpoints: transaminase elevation, steatosis indices, fibrosis markers, and cirrhosis mortality at sub-renal exposure levels across US, Korean, Thai, Chinese, and European cohorts (Table 3). Lead, stored predominantly in bone with moderate, mobilizable hepatic retention, shows dose-dependent hepatic enzyme associations in occupational cohorts but a more episodic hepatic signature. Mercury’s hepatic evidence is the thinnest, constrained by neurotropic targeting and the absence of speciation-stratified thresholds (Section 3.4.3). These differences caution against generalizing “heavy metal hepatotoxicity” as a single phenomenon.

Regarding threshold dissociation, current evidence raises the possibility that hepatic biomarker alterations may be detectable below conventional renal or neurological thresholds, particularly for Cd. However, these observations derive from cross-sectional and limited longitudinal studies with incomplete confounder adjustment; they do not constitute established liver-specific toxicity thresholds, and the modest absolute magnitude of reported transaminase elevations (Section 3.3) leaves open the possibility of subclinical or clinically insignificant change.

Strength of evidence. Established: metal transport into hepatocytes via DMT1, ZIP8/14, and speciation-dependent routes; mitochondrial disruption, Nrf2 suppression, and NF-κB/MAPK activation in experimental systems; Cd classification as a Group 1 carcinogen. Probable but incompletely demonstrated: hepatic effects at sub-renal Cd exposure in humans; dose-response consistency across cohorts. Hypothetical: metal-specific contributions to distinct MASLD stages; protective efficacy of plant foods in metal-exposed humans; speciation-stratified Hg hepatic thresholds; gut–liver axis amplification in humans.

### 6.2. Clinical and Public Health Implications

Given the limited clinical evidence in metal-exposed populations, plant-based dietary strategies should not be interpreted as established interventions for reducing metal-associated liver disease risk. Rather, plant-rich dietary patterns may be considered for their established general metabolic, cardiovascular, and liver health benefits, independent of metal exposure. Their potential protective effects against metal-induced liver injury remain mechanistically plausible hypotheses that require validation in appropriately designed human studies.

Emerging clinical and translational studies in MASLD indicate that plant food-based interventions may improve hepatic inflammation [134], insulin sensitivity, and lipid metabolism [135]. The translational evidence base comprises: cross-sectional and cohort associations between metal burden and liver enzymes in NHANES and Asian mining populations [18]; case-control associations between concurrent Pb/Cd exposure and type 2 diabetes in Thai mining areas [41]; dietary intake studies linking high phytochemical consumption with lower inflammatory markers and improved glycemic control in Thai health-promoting hospital settings [131]; prospective observational data associating polyphenol-rich diets with lower circulating metal concentrations in US and European cohorts [78]; and clinical trials and meta-analyses in MASLD populations (not metal-exposed populations) showing improved liver enzymes and insulin sensitivity with phytochemical supple-mentation [136]. In vitro studies in metal-exposed HepG2 models provide mechanistic support only [23,131]. Given the translational limitations established in Section 5.2, the dietary strategies discussed here should be understood as an unvalidated, adjunctive, experimental approach motivated by mechanistic plausibility rather than evidence-based clinical guidance, and must not be presented as a substitute for environmental and occupational exposure reduction, the only intervention in this review with direct evidence of reducing hepatic risk. Integrating environmental heavy metal exposure assessment into metabolic liver disease frameworks represents a research and surveillance priority [137,138,139]. Patients with MASLD in contaminated areas may be reasonable candidates for exposure assessment within appropriately designed research, and dietary counseling emphasizing cruciferous vegetables, colored vegetables, whole grains, legumes, and herbs/spices can be offered as a generally health-promoting pattern consistent with established nutritional guidelines, not as a validated treatment for metal-induced liver injury specifically [140,141].

Public health implementation should sequence interventions by strength of evidence, placing source control ahead of dietary strategies: (i) community-based programs promoting locally available plant foods as a complementary wellness measure; (ii) integrating exposure assessment into primary healthcare for surveillance and referral; (iii) agricultural support for low-metal crop production alongside dietary diversification; (iv) school-based nutrition education framed around general health; and (v) occupational health interventions providing dietary guidance as one component of a broader protection program including exposure monitoring and engineering controls. Reduction of heavy metal exposure at the source remains the primary and only evidence-supported preventive strategy discussed in this review, including remediation of contaminated soil and water, regulation of industrial and mining emissions, enforcement of occupational standards, and restriction of cadmium in fertilizers. Dietary and phytochemical approaches must not substitute for structural exposure control and should be communicated to communities, clinicians, and policymakers exclusively as complementary, unvalidated measures.

### 6.3. Forward-Looking Perspectives and Emerging Frameworks

Several emerging frameworks will shape the next phase of this field. The exposome framework integrates external exposures (environmental metals, diet, occupation) with internal biological layers (metabolomic and proteomic signatures of metal effects), enabling cumulative exposure assessment beyond single-biomarker approaches [125]. Multi-omics integration of transcriptomics, proteomics, metabolomics, and epigenomics can dissect which of the experimentally characterized pathways (Section 4.2, Section 4.3, Section 4.4 and Section 4.5) operate at environmentally relevant doses in humans. Single-cell RNA sequencing and spatial transcriptomics of metal-exposed liver tissue can resolve cell-type-specific responses among hepatocytes, Kupffer cells, stellate cells, and LSECs and map zonal injury patterns that bulk analyses obscure. Metagenomic profiling of the gut microbiome in chronically exposed populations can test the gut–liver axis hypotheses in Section 4.4 and Table 5. Finally, sex-stratified analyses and genotype-exposure interaction studies (e.g., metallothionein and transporter polymorphisms) are needed to explain the female susceptibility patterns reported in Korean and Chinese cohorts and the population-specific accumulation differences described in Section 3.2.

### 6.4. Future Research Priorities

Future priorities include: (i) human randomized controlled trials of plant food interventions in metal-exposed populations, ideally prestratified by mercury speciation profile to test whether speciation modifies dietary efficacy; (ii) prospective cohort studies incorporating repeated Cd biomarker measurements, advanced liver imaging, and mixture exposure modeling; (iii) multi-metal mixture investigations using BKMR powered specifically for three-way Cd × Pb × Hg interaction terms, which no published study has yet adequately estimated; (iv) development and validation of novel hepatic biomarkers (miR-122, GLDH, CK-18, elastography, MRI-PDFF) benchmarked against standard liver enzymes for detecting subclinical injury below the conventional renal threshold; (v) sex-stratified longitudinal surveillance to formally test the female susceptibility pattern reported in Korean and Chinese cohorts; (vi) gut microbiome–liver axis studies in chronically exposed populations; and (vii) exposome-based surveillance, multi-omics integration, single-cell transcriptomics of metal-exposed liver tissue, and spatial transcriptomics for zonal injury mapping [142].

## 7. Conclusions

This review synthesizes available evidence suggesting that heavy metal-induced hepatic inflammation represents a previously underappreciated public health consideration distinct from classical organ-specific toxicity. Taken together with the public health hierarchy established in Section 6.2, this evidence supports source-control prioritization as an actionable step, while liver-specific surveillance remains a potential future strategy requiring validated hepatic thresholds and biomarkers. The liver’s comparative sensitivity to chronic low-dose metal accumulation as a hypothesis for prospective testing rather than an established finding. Cadmium is associated with hepatic effects at sub-renal exposure levels, with dose-response patterns reported across diverse populations. However, this stops short of a proven causal effect given the observational nature of the underlying human studies. Cadmium, lead, and mercury trigger multifactorial dysfunction in experimental systems through the coupled mitochondrial, Nrf2, and NF-κB/MAPK cascades, supplemented by emerging pathways involving regulated cell death and the gut–liver axis, with cross-sectional human studies supporting associations with MASLD progression and increased HCC risk. Current exposure limits, primarily based on conventional renal and neurological endpoints, may not fully capture potential hepatic effects observed in cross-sectional studies; however, any revision of regulatory thresholds would require prospective confirmation that is not yet available. Plant-derived bioactives remain, on current evidence, mechanistically plausible candidates rather than validated interventions: preclinical multi-target activity has not been confirmed in metal-exposed human populations, and dietary advice should be framed as general health promotion rather than metal-specific hepatoprotection.

## Figures and Tables

**Table 1:** Environmental Sources, Toxicokinetics, and Hepatic Accumulation of Heavy Metals with Evidence Levels.

Heavy Metal	Primary Environmental Sources	Biological Half-Life (Hepatic)	Principal Transporters	Intracellular Targets	Conventional Targets (Kidney/Bone)	Evidence Level
Cadmium (Cd)	Mining tailings, phosphate fertilizers, cigarette smoke	10–30 years (long)	DMT1, ZIP8, ZIP14, TRPV6	Mitochondria, lysosomes, metallothionein complexes	Nephrotoxicity (glomerular/tubular damage); bone disease (decreased BMD, osteoporosis)	Experimental
Lead (Pb)	Paint, drinking water, ammunition, mining	Moderate; predominantly bone-stored, mobilizable	DMT1; voltage-gated Ca^2+^ channels (secondary)	Mitochondria (Ca^2+^ handling); hepatic proteins	Neurotoxicity (cognitive/developmental effects); secondary nephrotoxicity and bone accumulation	Experimental
Mercury (Hg)	Fish, dental amalgam, artisanal mining, industrial emissions	Speciation-dependent; hepatic levels far lower than kidney/brain for methylmercury	DMT1 (inorganic Hg); LAT1, cysteine transporters (methylmercury)	Mitochondria; glutathione-Hg complexes	Neurotoxicity (primary regulatory endpoint); secondary renal and neurological effects	Experimental

Hepatic accumulation characteristics are derived primarily from experimental animal models and vary markedly by metal; generalizations across metals should be made with caution [54]. Abbreviations: Cd = cadmium; Pb = lead; Hg = mercury; BMD = bone mineral density.

**Table 3:** Summary of Key Human Studies and Evidence Quality: Cadmium Exposure Levels, Hepatic Effects, Confounder Adjustment, and Limitations.

Study Population	Exposure Metric/Level	Liver Effect	Effect Size (95% CI)	Adjusted Confounders	Co- Exposure	Major Limitations	Evidence Quality
US Adults (NHANES) [60]	UCd top quartile (≈>0.50 μg/g cr)	Elevated ALT/AST; hepatic steatosis (HSI ≥ 36); liver-disease mortality	ALT (men): OR 2.21 (95% CI: 1.64–3.00); ALT (women): OR 1.26 (95% CI: 1.01–1.57); HSI ≥ 36: AOR 1.26 (95% CI: 1.11–1.43); liver-disease mortality: HR 3.42 (95% CI: 1.12–10.47)	BMI: yes; alcohol: partial; smoking: partial; viral hepatitis: no; Fe/Zn: no	Cd-dominant WQS weight for MASLD; Cd × Pb supra-additive (BKMR)	Cross-sectional for enzymes/steatosis; no hepatitis adjustment; biomarker reflects recent/cumulative exposure, not hepatic burden	Observational; moderate
Korean Adults (Community Cohort) [71]	BCd > 1.0 μg/L (top quartile vs. lowest)	Hepatic dysfunction; incident fatty liver over 10 y	BCd and hepatic fibrosis (FIB-4 > 2.67) in women: OR 1.668 (*p* = 0.012, CI not reported); ALD (highest vs. lowest BCd quartile): OR 1.68 (95% CI: 1.32–2.14)	BMI: yes; alcohol: no; hepatitis: partial; smoking: no; Fe/Zn: no	Mixture-adjusted reanalysis not identified	Cross-sectional component; limited confounder set	Observational; moderate
Thai Mining Communities [42]	UCd 1.0–3.5 μg/g cr	Transaminase elevation	Adjusted OR 2.59 (95% CI: 1.06–2.50);	BMI: yes; alcohol: yes; hepatitis: no; smoking: no; Fe/Zn: partial	Concurrent Pb; supra-additive interaction reported	No viral hepatitis adjustment; Hg not bio-monitored	Observational; moderate
Chinese Industrial Workers [5]	BCd 2.0–4.0 μg/L	Progressive GGT elevation	Amplified in smokers; quantitative OR 2.53 (95% CI: 2.01–2.60);	Smoking: yes; BMI: no; alcohol: no; hepatitis: no	As/Pb co-exposure amplifies risk	Occupational co-exposures unmeasured; no metabolic adjustment	Observational; low–moderate
Multi-Cohort Fibrosis Analysis [17]	UCd < 5.0 μg/g cr (sub-renal)	FIB-4, NFS fibrosis markers	FIB-4 ≥ 2.67: AOR 1.73 (95% CI: 1.09–2.87)	BMI: partial; alcohol: partial; hepatitis: no	Stratified mixture estimates not reported	Heterogeneous cohorts; cross-sectional	Observational; low–moderate
European Adults (EFSA Biomonitoring) [40]	BCd > 0.80 μg/L	MASLD progression	≈1.8× increased odds reported	BMI: yes; alcohol: no; hepatitis: no; Fe/Zn: partial	Multi-metal dietary exposure not modeled	Hg co-exposure from fish not disentangled	Observational; moderate
Cirrhosis-Mortality Cohort [72]	Highest UCd quartile vs. lowest	Cirrhosis-related mortality	Liver cirrhosis mortality: HR 2.05 (95% CI: 0.59–7.09)	Alcohol: yes; hepatitis: yes; BMI: yes; smoking: partial		Best-adjusted; residual dietary/occupational confounding possible	Observational, prospective; moderate–high

UCd, urinary cadmium; BCd, blood cadmium; cr, creatinine; GGT, gamma-glutamyl transferase; FIB-4, Fibrosis-4 index; NFS, NAFLD fibrosis score (retained in the instrument name); HR, hazard ratio; OR, odds ratio; WQS, weighted quantile sum regression; BKMR, Bayesian kernel machine regression. Where exact 95% confidence intervals or fully adjusted effect sizes were not reported in the primary sources, this is explicitly noted in the table. No single study simultaneously adjusted for the full set of plausible confounders (BMI, alcohol, smoking, viral hepatitis, iron/zinc status, and co-exposures); the associations should therefore be interpreted as upper-bound estimates of any true metal-specific effect and as population-specific observations rather than universal thresholds.

**Table 4:** Inflammation-Related Hepatic Effects of Heavy Metals and Potential Links to Hepatocellular Carcinoma (HCC) with Level of Evidence.

Heavy Metal	Carcinogenic Classification (IARC)	Dominant Hepatic Effects	Key Molecular Pathways	Relevance to HCC	Evidence Level
Cadmium (Cd)	Group 1	Genotoxicity via ROS-induced DNA damage; epigenetic silencing; chronic NF-κB/IL-6 inflammation	ROS/Nrf2 suppression; NF-κB/IL-6 axis; PI3K/AKT Activation	Promotes hepatocyte proliferation and immune escape; associated with elevated liver enzymes and steatosis in exposed cohorts [11,101,123,124]	Experimental + Observational
Lead (Pb)	Group 2A	Oxidative DNA strand breaks; base excision repair inhibition; hepatic stellate cell activation	ERK/p38 MAPK; TGF-β signaling	Drives fibrogenesis and pro-tumorigenic microenvironment; elevated blood Pb correlates with transaminase elevation in mining workers [84,99,104,105]	Experimental + Observational
Mercury (Hg)	Group 2B	Immunosuppression; chronic inflammation; oxidative DNA damage	Thiol-binding enzyme inhibition; ROS accumulation	Data insufficient to establish HCC risk magnitude [98,121,122]	Experimental—human data sparse

Carcinogenic classifications per IARC Monographs. Mechanistic pathways summarized from experimental models; human epidemiological evidence remains largely associative and subject to confounding. Abbreviations: ROS, reactive oxygen species; IARC, International Agency for Research on Cancer.

**Table 5:** Multi-Target Hepatoprotective Mechanisms of Plant-Derived Bioactives: Pathways, Food Sources, and Levels of Evidence.

Signaling Pathway/Food Category	Metal-Induced Dysfunction Targeted	Mechanism of Protection	Key Bioactive Compounds/Food Sources	Functional Outcome	Evidence Level
Mitochondrial function	ETC disruption, ROS increase, ATP decrease	Stabilization via terpenoid-mediated AMPK activation	Triterpenes, sesquiterpenes	Restored bioenergetics, reduced oxidative stress [119,132]	Experimental
Nrf2 antioxidant response	Metal-induced Nrf2 degradation; antioxidant gene suppression	Keap1 modification preventing ubiquitination; sustained Nrf2 activation	Polyphenols (resveratrol, curcumin), flavonoids	Enhanced SOD, catalase, GCL, NQO1 [101,124]	Experimental
NF-κB signaling	IκBα degradation; chronic pro-inflammatory state	IκBα stabilization; direct IKK inhibition; ROS scavenging	Polyphenols, flavonoids	Decreased TNF-α, IL-6, IL-1β [23,58]	Experimental
MAPK cascades	JNK/p38/ERK activation; inflammatory gene elevation	Pathway inhibition via ROS reduction and kinase modulation	Terpenoids, polyphenols, alkaloids	Reduced inflammatory signaling amplification [105,133]	Experimental
Cruciferous vegetables (broccoli, Brussels sprouts, cabbage, kale)	Reduced phase II detoxification capacity; metal bioavailability	Glucosinolates/sulforaphane/indole-3-carbinol-driven phase II enzyme induction (GST, NQO1)	Glucosinolates, sulforaphane, indole-3-carbinol	Enhanced GST expression; improved detoxification capacity in observational cohorts; Nrf2/ARE activation and reduced oxidative stress in rat models	Observational + Experimental
Colored vegetables (spinach, kale, carrots, tomatoes, peppers)	Oxidative and inflammatory burden	Carotenoid/polyphenol-mediated ROS scavenging	Carotenoids, polyphenols	Lower circulating metal levels; improved antioxidant status [Observational, [72,131]]; attenuated lipid peroxidation [Experimental]	Observational + Experimental
Berries (blueberries, raspberries, strawberries, cranberries)	Oxidative stress responses and mitochondrial preservation	Anthocyanin/resveratrol/ellagic acid/quercetin-mediated redox modulation	Anthocyanins, resveratrol, ellagic acid, quercetin	Reduced inflammatory markers in cohort studies [Observational]; attenuated ROS/NF-κB activation in HepG2 models [Experimental; [71,127,128]]	Observational + Experimental
Green tea (Camellia sinensis)	Integrated redox and metabolic dysregulation	Catechin (EGCG, ECG)/L-theanine-mediated redox regulation via AMPK	Catechins, L-theanine	Inverse association with liver enzyme elevation [Observational, [128,130]]; improved mitochondrial respiration in animal models [Experimental]	Observational + Experimental
Herbs and spices (turmeric, garlic, ginger)	Inflammatory signaling, antioxidant defenses, metal bioavailability	Curcumin/allicin/gingerol-mediated modulation	Curcumin, allicin, gingerol	Reduced inflammatory markers [128]; enhanced metal chelation in animal models [Experimental]	Experimental
Legumes (beans, lentils, chickpeas, peas)	Hepatic metal accumulation; glycemic/metabolic dysregulation	Phytate-mediated metal chelation; AMPK-mediated metabolic regulation	Flavonoids, saponins, phytic acid	Improved glycemic control; reduced diabetes risk [Observational, [131]]; reduced hepatic lipid accumulation [Experimental]	Observational + Experimental
Whole grains (brown rice, oats, barley, whole wheat)	Gut microbiota dysregulation; impaired metal excretion	Fiber/β-glucan-mediated gut microbiota modulation and enhanced metal excretion	Phenolic compounds, fiber, β-glucans	Linked to lower inflammatory markers [Observational, [17]]; improved gut-liver axis function in animal models	Observational + Experimental

Mechanistic evidence derives primarily from in vitro and rodent models; no dedicated randomized controlled trials have evaluated these compounds in metal-exposed human populations. Abbreviations: AMPK, adenosine monophosphate-activated protein kinase; ETC, electron transport chain; GST, glutathione S-transferase; ARE, antioxidant response element.

## Data Availability

No new data were created or analyzed in this study. Data sharing is not applicable to this article.
